# Energy Beyond Food: Foraging Theory Informs Time Spent in Thermals by a Large Soaring Bird

**DOI:** 10.1371/journal.pone.0027375

**Published:** 2011-11-08

**Authors:** Emily L. C. Shepard, Sergio A. Lambertucci, Diego Vallmitjana, Rory P. Wilson

**Affiliations:** 1 Department of Biosciences, College of Science, Swansea University, Swansea, United Kingdom; 2 Laboratorio Ecotono, Universidad Nacional del Comahue - INIBIOMA CONICET, Bariloche, Río Negro, Argentina; University of Western Ontario, Canada

## Abstract

Current understanding of how animals search for and exploit food resources is based on microeconomic models. Although widely used to examine feeding, such constructs should inform other energy-harvesting situations where theoretical assumptions are met. In fact, some animals extract non-food forms of energy from the environment, such as birds that soar in updraughts. This study examined whether the gains in potential energy (altitude) followed efficiency-maximising predictions in the world's heaviest soaring bird, the Andean condor (*Vultur gryphus*). Animal-attached technology was used to record condor flight paths in three-dimensions. Tracks showed that time spent in patchy thermals was broadly consistent with a strategy to maximise the rate of potential energy gain. However, the rate of climb just prior to leaving a thermal increased with thermal strength and exit altitude. This suggests higher rates of energetic gain may not be advantageous where the resulting gain in altitude would lead to a reduction in the ability to search the ground for food. Consequently, soaring behaviour appeared to be modulated by the need to reconcile differing potential energy and food energy distributions. We suggest that foraging constructs may provide insight into the exploitation of non-food energy forms, and that non-food energy distributions may be more important in informing patterns of movement and residency over a range of scales than previously considered.

## Introduction

Microeconomic models have been fundamental to the development of foraging theory, in particular, ‘patch-use’ models, which predict patterns of residency in, and travel between, patchily distributed food resources [1], [2]. Many predictions of patch models have been shown to hold true, at least qualitatively, for a large range of animals and foraging scenarios [2], and consequently these models have far-reaching implications for our understanding of the way animals move with respect to the distribution of food resources. Though widely used to examine feeding, such microeconomic models should inform other energy-harvesting situations where theoretical assumptions are met.

Soaring birds are a clear case of animals that harvest a non-food form of energy from the environment. These birds can exploit kinetic energy available in convective updraughts, or ‘thermals’, to gain altitude and glide to another location, reducing the need for flapping flight and consequently, the costs of horizontal travel [3], [4], [5]. The gain in potential energy (a linear function of altitude assuming constant mass) is proportional to the horizontal distance a bird is able to glide, assuming the speed of travel between thermals is constant [3]. Two physical features of updraughts qualify them as ‘patches’ of energy analogous to those used in foraging theory: firstly, thermals are formed principally by differential heating of the underlying topography and/or substrate, giving them a spatially variable distribution [6], secondly the rate of potential energy gain within thermals eventually declines with height gained. This follows from the standard decrease in temperature with altitude, which, in simplified terms, reduces the rate at which a warm body of air rises [6], exposing soaring birds to diminishing energy returns and a patch-leaving decision scenario. Although in reality the vertical velocity may not be evenly related to altitude (as for example thermals may rise as detached vortex rings [6]), a bird climbing within a thermal will eventually experience diminishing returns. Consequently, we propose that soaring birds should transit between patches in a manner analogous to animals exploiting heterogeneous food resources.

Birds can increase their energy gain from updraughts by reducing their flight speed through them [7]. While small birds may benefit from updraughts by reducing their speed but keeping a straight flight path through them (‘straight-line soaring’), large birds such as vultures may reap higher energetic rewards by circling to maintain their position in a core area of maximum lift [4]. Consequently, for large birds, the decision to remain in a patch is associated with a particular movement signature that should be readily identifiable in movement data, given sufficient resolution.

This study examines the harvesting of potential-energy in the world's heaviest soaring bird, the Andean condor (*Vultur gryphus*) [8], using animal-attached technology to define their three-dimensional flight paths at a fine-scale. The logistical difficulties associated with the collection of such data [3] have, to date, precluded quantitative examination of fine-scale movement paths in free-living birds. Andean condors are predicted to experience among the highest costs of flapping flight [9], making them dependent upon environmentally-generated lift to cover the distances necessary to search for food [10]. In fact, Andean condors may not even be capable of maintaining altitude through flapping flight alone [10] and therefore must have exceptional soaring capabilities, making them ideal model organisms for the study of soaring strategies. We hypothesised that time spent in thermal updraughts would not be random, but rather conform to a strategy that increases the efficiency with which they harvest this energy form, such as minimising the overall energy expended or maximising the rate of horizontal travel [4], [11]. In the latter scenario, birds would be expected to leave a thermal when their vertical velocity (equivalent to the rate of energy gain) declined to the overall average (i.e. the marginal value) for that habitat [1]. In an energy-minimising scenario, birds would be predicted to remain in thermals while they experienced a positive net vertical velocity.

## Methods

### Device deployment

Five adult female condors were captured in October 2010 in northwest Argentine Patagonia with baited cannon net traps, held in the shade while morphometric data were collected and loggers were fitted, and then released at the same location. Permissions to capture, tag and/or monitor condors were provided by Dirección de Fauna Silvestre de Río Negro, the Argentine National Park Administration, and the owners and managers of local farms. Procedures were also approved by the ethics committee of Swansea University (approval numbers were not applicable as research was not covered by the UK's Animals and Scientific Procedures Act 1986).

Loggers were enclosed in a streamlined and lightweight, black plastic housing (total mass 135 g: 1.3% of mean female body mass) and mounted on the upper back via a base-plate taped to the feathers. The housing contained a Daily Diary (DD) with 22 bit resolution [12], a GPS logger (iGotU GT-600, Mobile Action Technology), and a VHF transmitter (BD-2, Holohil Systems Ltd.) to aid device recovery. The DD recorded barometric pressure (thereby altitude), compass heading, and triaxial acceleration (to count wingbeats) at a frequency of 6 Hz. The GPS was set to record once every 11 s. Birds were also fitted with patagial PTT tags (Microwave Telemetry Inc., total mass 50 g). Resulting positional data showed that females maintained similar patterns of area use for four months following tagging (S Lambertucci et al. unpubl. data). One DD unit was recovered by re-trapping; other birds have since been sighted without DDs, designed to drop off after ca. 1 month.

### Derivation of flight paths

Barometric pressure data were converted into absolute altitude by regressing mean values of pressure against mean GPS-derived height 15 minutes prior-to and following each flight to a new roost. Separate calibrations were performed for each day. Altitude values were smoothed with a running mean over the 2 s (12 data points) preceding each value to remove the step functions arising where the 1 m sensor resolution was exceeded.

Values of compass heading were combined with the mean GPS groundspeed to produce an initial dead-reckoned flight path [13] originating from known start coordinates. The difference between the dead-reckoned and known coordinates at subsequent GPS locations was then used to derive a correction factor for the dead-reckoned track, assuming drift to be constant over time [13]. Altitude was taken from the calibrated DD data.

### Thermal residence time

Circling flight maintains a bird's position within a thermal and was therefore taken to represent the decision to remain in a patch. The beginning and end of patch exploitation was readily identifiable in the compass heading as points of inflection between straight-line and circular motion. The degree to which the observed patch time was predicted by the time where the instantaneous vertical velocity exceeded the mean overall vertical velocity, was examined using a simple regression analysis (as the three flights were from one individual (see Results), and the numbers of thermal patches were unbalanced between flights, with each of the three flights used for the analysis containing 11, 3 and 2 thermal climbs).

The vertical velocity (which, in this study, corresponds to the rate of energy gain) was taken as altitude at time t + 1 s less that at time t. These values were used to calculate i) the mean vertical velocity from the beginning of the flight to time T (µ*V*_z_) and ii) the instantaneous vertical velocity (*V*_z_), where values were smoothed with an FFT filter over 10 s to reduce fine-scale variability. Filtering operations were performed in OriginLab.

Patch residence times were examined for all but the last thermal from each flight, where patch-leaving decisions may have been influenced by landing altitude, rather than the energy gain alone. Kolmogorov-Smirnov tests and linear regressions were performed in Minitab 14.

## Results

**Figure 1:**
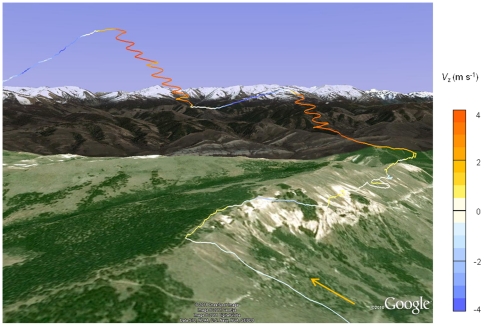
Condor 3-dimensional flight path. A section of a condor flight path shown in Google Earth, with the orange arrow indicating the direction of travel. The vertical velocity, shown in colour, was at a maximum while the bird was circling and was still positive on departure from thermals.

**Figure 2:**
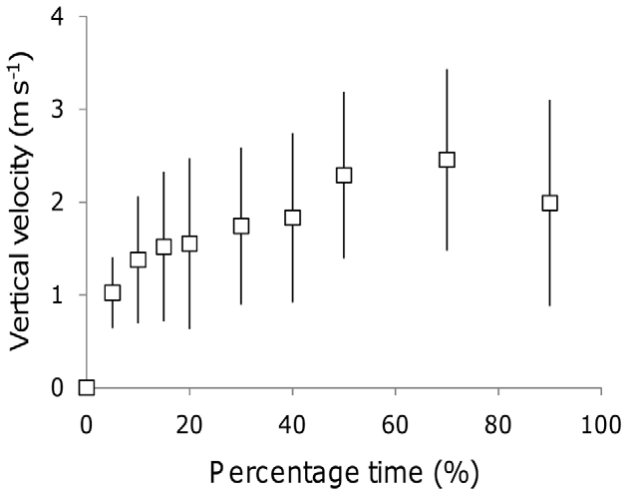
The rate of potential energy gain in thermals. Vertical velocity (*V*_z_) experienced by an Andean condor during 16 thermals (± SD) as a function of time from the initiation of positive rates of climb to the bird's departure from the thermal. Each *V*_z_ value was calculated as the average over a 3 s period for the corresponding % time value.

Data from a single Daily Diary, providing XYZ location data at a frequency of 6 Hz over a five day period, showed that only 4.6% of the total time was spent on the wing, in fifty separate flights ranging from 0.2–72.3 minutes. Three of these flights resulted in marked horizontal displacement, where distances of 22.4, 65.1 and 26.0 km (given as the sum distance between GPS locations) were covered in 31.7, 72.3 and 34.9 minutes, respectively. These ‘transit’ flights were characterised by a infrequent and isolated wingbeats (outside periods of take-off and landing), and clear, repetitive circling behaviour (Fig. 1). Once the bird began to circle within a thermal, it experienced a rapid initial increase in *V*_z_, followed by a decline in *V*_z_ prior to the bird leaving the patch (Fig. 2).

**Figure 3:**
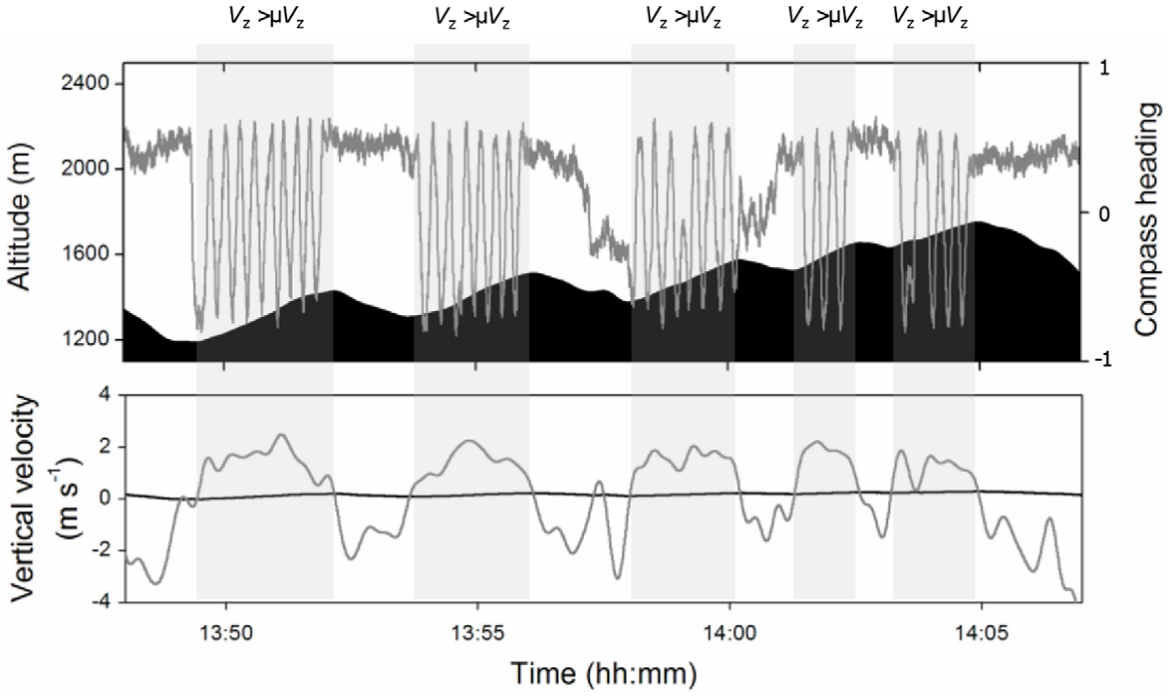
The occurrence of circling flight. DD-derived values of compass heading (dark grey line) and flight altitude (solid black) are shown in the upper panel during five consecutive periods of climb. The wave-form pattern in the compass heading is indicative of circling behaviour, with each sine-wave representing a full turn, reaching a maximum value when the bird faced north. *V*_z_ is shown in the lower panel for the same period. Circling occurred where values of *V*_z_ (grey line) exceeded the mean overall vertical velocity (µ*V*_z_, black line), these periods are shaded in grey.

During the three transit flights, it was assumed that sequences of soaring and gliding were undertaken for horizontal transport. Visual inspection of circling behaviour within these flights suggested that the bird remained in thermals until the rates of altitude gain had declined to similar levels of profitability (Fig. 3). Indeed, analysis of the time spent circling showed that it was significantly predicted by the time period that the instantaneous vertical velocity (*V*_z_) exceeded the mean overall vertical velocity (µ*V*_z_) (time spent circling ** = ** 0.92*x* + 0.6: R^2^ = 86.3%, F = 88.5, P<0.001, N = 3 flights Fig. 3).

**Figure 4:**
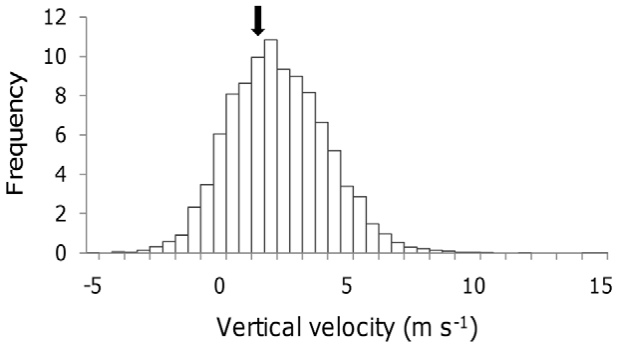
The frequency of vertical velocities experienced in thermals. Unsmoothed *V*_z_ values during circling behaviour (the mean *V*_z_ on departure from thermals is indicated by a black arrow). Note the x-axis range has been reduced for clarity, where the full range was −21.4 to 17.5 m s^−1^.

**Figure 5:**
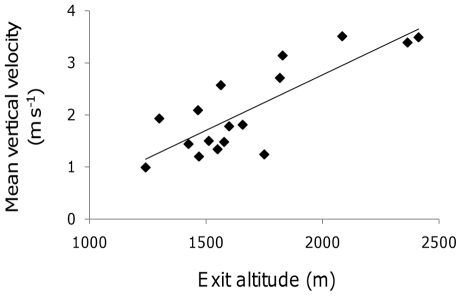
Climb rate and altitude on exit from thermals. The mean *V*_z_ experienced per thermal was positively related to the altitude at which the bird left the thermal.

Further analysis showed that the time spent in thermals was not correlated with the mean *V*_z_ encountered within them (R^2^ = 0.0), i.e. patch quality. However the mean *V*_z_ in a thermal was positively related to the exit altitude (R^2^ = 64.2, F = 25.1, P<0.01, N = 3 flights). The values of *V*_z_ on departure from a patch (mean 1.34 m s^−1^, SD ± 0.98, Fig. 4) also increased with exit altitude (R^2^ = 35.9, F = 7.8, P = 0.01, and R^2^ = 64.2, F = 25.1, P<0.01, N = 3 flights, Fig. 5).

Values of body mass for female condors ranged from 10.0 – 11.32 kg. The mass of the bird for which the DD was recovered was 11.32 kg.

## Discussion

This study presents precise data on the fine-scale, three-dimensional flight paths and behaviour of a free-living bird. These data, which are, to the best of our knowledge, the first such data derived from a free-living bird using animal-attached technology [3], [5], [9], [14], enabled the quantitative examination of rates of potential energy gain and time spent in thermal updraughts over a five day period. The pattern of energy gain within thermals was comparable to the acquisition of food resources, in that, on entering a patch, the bird experienced a rapid increase in vertical velocity (*V*_z_, here equivalent to the rate of energy gain), which declined with time in the thermal (Fig. 2). The dominant paradigm in predicting patterns of patch use is the Marginal Value Theorem (MVT) [1], which states that animals foraging on patchy resources should leave a patch when the capture rate declines to the average capture rate for the habitat in order to maximise their net rate of energy gain. Our results, though limited to a few flights, show that time spent in thermal patches was strongly predicted by the time where the instantaneous *V*_z_ exceeded the overall mean; consistent with marginal value predictions [1]. In addition, the bird left thermals while still experiencing positive, and appreciable, rates of climb (mean 1.34 m s^−1^), which is not consistent with a strategy to maximise the gross energy gain.

Another prediction of the MVT is that animals should spend more time in a patch when the travel time between patches is high [1]. This prediction is difficult to test as the travel speed over ground is a function of the wind speed and direction (not measured in the present study) as well as inter-patch distance. Previous findings by Akos et al [3] showed that peregrine falcons (*Falco peregrinus*) select inter-thermal glide speeds that maximise the rate of horizontal travel (which would be consistent with maximising the overall rate of energy gain), however speed was not considered in relation to time in thermals. Future research with a larger dataset may be able to quantify the influence of inter-patch travel time on patch residence time.

In fact, for the condor, the patch-leaving decision cannot be explained by marginal value criteria alone, as the *V*_z_ on exit from thermals increased with the mean strength of the updraughts. This goes against a further prediction of the MVT, which states that birds should spend longer exploiting patches of higher quality [15], [16], reducing all patches to the same level of profitability. Indeed, here, there was no correlation between the mean *V*_z_ in a thermal and patch time. Furthermore, the giving-up threshold increased with the altitude at which the bird exited a thermal. In this study it was assumed that the long flights were undertaken for horizontal transport, as they represented directed movement between distinct roost sites. However, given that the bird was not migrating, it is likely that it was also searching for food resources. For scavenging birds, such as condors, the ability to search for carrion (comprised mainly of medium to large herbivores [17]) varies with height above the ground [18], and consequently any selection pressure to increase their energetic gains from thermal updraughts is likely to be modulated by the need to search for food energy resources. Any initial increases in search efficiency with altitude, due to expansion of the searchable area, will be modulated by the bird's ability to detect food within that area, which will decline with altitude. This may explain why the exit *V*_z_ increased with the exit altitude, as, above a certain altitude, positive rates of energy gain may no longer be advantageous. This threshold level in altitude is likely to vary with respect to factors including height above ground level, substrate, carrion type and density, and ability to perceive other scavengers moving towards the food source [18].

Other currencies, including the distribution of predation risk, are known to modulate the way animals exploit energy resources [19], [20], yet these effects are distinct from the need for condors to exploit sources of potential energy while simultaneously searching for carrion. How soaring birds reconcile differences between the distributions of updraughts and food is poorly understood, although the availability of updraughts is likely to be a key determinant of the energetic costs of accessing food resources in various locations [21]. It may be that this has received relatively little attention as data on the flight trajectories of soaring birds are gathered most frequently during migration [22], [23], [24], [25], as opposed to foraging trips.

Condors lie at the extreme end of the spectrum in terms of their reliance on environmentally-generated lift, yet a wide range of flying animals are likely to experience selective pressure to exploit these energy sources efficiently, due to the substantial energetic gains available in a range of lift-producing systems. Updraughts in thermals are typically in the order of 1 –5 m s^−1^ and the gain in potential energy can be found from 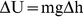
(1)where U is potential energy (J), m is the mass of the object (kg), g the acceleration due to gravity (m s^−2^) and h the altitude gain (m). The mean climb rate recorded during circling behaviour was 2.1 m s^−1^, and the maximum (smoothed value) 14.8 m s^−1^, which would have yielded potential energy gains of 233 and 1632 J/s respectively (using the mass of 11.32 kg recorded for this female condor). Comparison of this with food energy gains in condors shows that the *ca*. 5900 kJ yielded from 600 g of meat provided daily to captive birds (G. Wiemeyer, pers. comm., assuming mutton assimilated with an efficiency of 80% [26]) would take 7 h to accrue in a thermal where the bird gained altitude at 2.1 m s^−1^ or 1 h in an updraught of 14.8 m s^−1^. The comparison is pertinent as gains in potential energy lead directly to calorific savings by obviating the need for powered flight [9]. During the flights documented here, the condor gained sufficient potential energy to offset losses between sources of rising air and remain airborne for 2.3 h with only infrequent and isolated wing beats. Clearly, the energetic gains will be proportionately larger for heavier birds, as the potential energy gain is proportional to bird mass, and avian metabolic rates increase as a decreasing function of mass [9], [27], yet birds as small as European bee-eaters (*Merops apiaster*), weighing 55 g, have recently been shown to switch from flapping to soaring-gliding flight in areas of high turbulent kinetic energy [7] (indicative of thermal intensity).

In conclusion, our results strongly suggest that the Andean condor exploits sources of potential energy in a non-random manner. The discrete nature of thermal updraughts and the diminishing returns experienced by birds exploiting them, mean that patch models can be used to provide insight into the currencies that influence the harvesting of potential energy in soaring birds. Although there is evidence that the time in thermals was influenced by a strategy to maximise the rate of energy gain, this appears to have been bounded by the need to search for food. If, as our results suggest, birds do employ efficiency-maximising strategies to harvest potential energy, their resulting patterns of area-use will represent a concatenated response to multiple resource distributions, which are likely to differ fundamentally in space and time. Soaring birds are not alone in harvesting non-food forms of energy from the environment: Wider examples include heat gain by basking reptiles [28] and kinetic energy gain by fish migrating in tidal systems [29]. Consequently, understanding the nature of non-food energy distributions may help explain patterns of movement and residence at a range of scales, particularly where animal movement is not directed at acquiring food. It may also add complexity to the analysis of animal search strategies, which are generally interpreted as a response to a single resource [30], [31].
